# Methicillin-resistant *Staphylococcus aureus* in Pig Farming

**DOI:** 10.3201/eid1112.050428

**Published:** 2005-12

**Authors:** Andreas Voss, Frans Loeffen, Judith Bakker, Corne Klaassen, Mireille Wulf

**Affiliations:** * Radboud University Medical Centre, Nijmegen, the Netherlands; † Canisius-Wilhelmina Hospital, Nijmegen, the Netherlands

**Keywords:** MRSA, pigs, farming, *spa*-typing, dispatch

## Abstract

We conducted a study among a group of 26 regional pig farmers to determine the methicillin-resistant *Staphylococcus aureus* prevalence rate and found it was >760 times greater than the rate of patients admitted to Dutch hospitals. While *spa*-type t108 is apparently a more widespread clone among pig farmers and their environment, we did find other *spa*-types.

Methicillin-resistant *Staphylococcus aureus* (MRSA) has become a major nosocomial pathogen, highly prevalent in many European countries and throughout the world (*1*). In the Netherlands, the prevalence of MRSA among clinical isolates is still <1%, among the lowest in Europe (*1*). This low prevalence is probably best explained by the national policy that entails strict screening and isolation of all persons who are considered at high risk for MRSA when admitted to a hospital. This high-risk population has essentially consisted of patients admitted to or treated in foreign hospitals. As a result of this policy for all healthcare institutions, the prevalence of MRSA in the Dutch community is extremely low as well. In a recent study among ≈10,000 patients admitted to 4 Dutch hospitals, 23% carried *S. aureus*, but only 0.03% of the isolates were methicillin-resistant (*2*).

In July 2004, we unexpectedly found MRSA in the preoperative screening cultures of a 6-month-old girl before thoracic surgery. Neither the girl nor her family (parents, 1 sister) had a history of traveling or admission to a foreign hospital. In the following months, the girl remained colonized with MRSA during consecutive decolonization attempts. Subsequently, the girl's parents were found to be positive for MRSA. The family lived on a farm and raised pigs.

To further investigate pig farming as a possible source of MRSA in Dutch patients, we screened a selection of pigs owned by the MRSA-positive farmer, and other regional pig farmers in November 2004. In January and February 2005, 2 new cases of MRSA were identified, one in a pig farmer from a different region and one in the son of a veterinarian who worked mostly with pigs. Subsequently, the strain was also isolated from the veterinarian and from a nurse in the hospital unit to which the son was admitted.

Although the aforementioned cases were unrelated in time and location, they shared some features. In all the cases, other family members were MRSA-positive, decolonization was repeatedly unsuccessful, and genotyping performed in the National Institute of Public Health and Environment (RIVM, Bilthoven, the Netherlands) showed the strains were not typeable by pulsed-field gel electrophoresis (PFGE) with restriction endonuclease *Sma*I (the standard method).

## The Study

Initially, the nares of 10 pigs were cultured. All were negative for MRSA. At a later stage, the perineum of 30 pigs was cultured; 1 was positive for MRSA. The regional pig farmers were screened (throat and nares) during a monthly professional meeting that happened to be on the farm of the MRSA-positive family, at the time of investigation. With the exception of this meeting, the farmers had no further epidemiologic links, other than being from the southeastern region of the Netherlands. Six (23%) of the 26 farmers were colonized with MRSA.

As mentioned above, all MRSA isolates were resistant to digestion with restriction-endonuclease *Sma*I, when typing with PFGE was attempted. To ensure that we did not falsely classify a pig-related staphylococcal species as MRSA, the identification of all isolates was confirmed by testing for the presence of a *S. aureus*–specific DNA element as well as the *MecA* gene, according to the methods of Reischl et al. (*3*). To compare the MRSA isolates, we performed random amplified polymorphic DNA analysis with primers Eric II (5´-AAG TAA GTG ACT GGG GTG AGC G-3´), RW3A (5´-TCG CTC AAA ACA ACG ACA CC-3´), D14307 (5´-GGT TGG GTG AGA ATT GCA CG-3´) and *spa*-typing.

Overall, 3 different MRSA strains were identified. The isolates of the girl (case-patient A), her parents, and the pig from their farm were identical with random amplified polymorphic DNA and belonged to *spa*-type t108. Furthermore, one of the regional pig farmers screened during the meeting, the pig farmer from a different region (case-patient B), the young boy (case-patient C), as well as his father and the nurse who treated the boy, were colonized with the same strain (Table). Three of the regional pig farmers shared *spa*-type 567. The isolate from the remaining MRSA-positive regional farmer showed a *spa*-type not previously described (Table).

**Table:** Molecular typing of methicillin-resistant Staphylococcus aureus isolates

Case-patients	Date of culture	Random amplified polymorphic DNA type	*Spa*-type
Patient A (girl)	Jul 2004	A	108
Regional farmer 1 (father of patient A)	Aug 2004	A	108
Mother of patient A	Nov 2004	A	108
Pig	Feb 2005	A	108
Patient B (farmer, different region)	Jan 2005	A	108
Patient C (boy)	Feb 2005	A	108
Father (veterinarian) of patient C	Feb 2005	A	108
Nurse of patient C	Feb 2005	A	108
Regional farmer 2	Nov 2004	Not done	108
Regional farmer 3	Nov 2004	Not done	567
Regional farmer 4	Nov 2004	Not done	567
Regional farmer 5	Nov 2004	Not done	567
Regional farmer 6	Nov 2004	Not done	943

## Conclusions

Recently, MRSA has been found in horses and in persons who take care of them (*4*). Human carriage has also been linked to colonized companion cats and dogs (*5**,**6*). While Lee et al. (*7*) reported an MRSA isolation frequency of 0.6% in major food animals, but did not find MRSA in 469 samples from pigs, Armand-Lefevre et al. (*8*) described *S. aureus* (methicillin-susceptible and -resistant) carriage among pigs and pig farmers. Although the authors showed that both farmers and pigs carried methicillin-sensitive *S. aureus* and MRSA and that both groups shared certain multilocus sequence typing, the isolates came from separate, nonrelated collections.

Here we demonstrate transmission of MRSA between an animal and human (pig and pig farmer), between family members (pig farmers and their families), and between a nurse and patient in the hospital. The unexpected high frequency of MRSA among the group of regional pig farmers (>760× higher than in the general Dutch population) indicates that their profession might put them at risk for MRSA colonization. Overall, we found 3 different MRSA strains, including a new *spa*-type. Therefore, we expect that multiple strains are present in the pig population and the pig farmers. The strain with *spa*-type t108 appears to be more prevalent and widespread, given that the strain spread from animal to human, between family members, between patient and nurse, and among pig farmers from different regions.

Further research on a larger scale is needed to see if these observations hold true in other regions. If so, pig farming poses a significant risk factor for MRSA carriage in humans that warrants screening wherever pig farmers or their family members are admitted to a hospital.
